# Citizen science across borders: employing action design research to identify and address digital skills challenges in the EU through collaborative solutions

**DOI:** 10.3389/fsoc.2025.1558618

**Published:** 2025-05-14

**Authors:** Valtins Karlis, Sarma Zane Emilija, Chiaki Sekiguchi Bems, Emily Kouzaridi, Karine Lan Hing Ting

**Affiliations:** ^1^International Education Research Center, Riga Technical University, Riga, Latvia; ^2^European University of Technology, Riga, Latvia; ^3^Cyprus University of Technology, Limassol, Cyprus; ^4^European University of Technology, Limassol, Cyprus; ^5^European University of Technology, Secretariat General, Troyes, France

**Keywords:** citizen science, digital skills, citizen lab, action design research, co-creation

## Abstract

The European University Alliance European University of Technology (EUt+) has endeavored to make a Citizen Lab, as part of the Horizon 2020 project “EXTRAS,” aimed to explore the use of Action Design Research (ADR) methodology for fostering citizen science across boarders and during one of the pilot activities focused on digital skills development. Interactions provided an international forum for educational practitioners from France, Cyprus, and Bulgaria, alongside researchers from the European University of Technology, to collaboratively address the challenges of digital upskilling and reskilling in the European context. Through a series of three workshops, participants engaged in meaningful discussions on the inclusion of diverse identities and intersectionality in digital skill development programs. The ADR framework guided the workshops, facilitating thematic clustering of ideas, generation of design principles, and the cocreation of potential solutions. The findings emphasized the importance of inclusive, identitysensitive approaches to digital skills education, with a focus on adult learners facing various barriers. The workshops were deemed successful in testing the applicability of ADR for citizen science in an international setting, serving as a proof of concept for future Citizen Lab endeavors in diverse contexts and subject areas. The study highlights the potential of Citizen Labs to generate practical and meaningful insights through participatory, cross-border collaboration.

## 1 Introduction

Digital skills are being discussed across the EU and as a responsible alliance of nine universities from nine different EU countries (France, Latvia, Germany, Cyprus, Spain, Bulgaria, Romania, Italy, and Ireland) this issue has also been addressed by the EUt+ - European University alliance (https://www.univ-tech.eu), established in 2019. Digital skills were selected as a point of focus for the Citizen Lab of EUt+ that undertakes citizen science activities. The idea and force behind the concept of citizen laboratories stem from Medialab-Prado (MLP) in Madrid (https://www.medialab-matadero.es/), that started in 2002 with a space dedicated to exchanging experiences and became fully operational in 2013. Its pioneer developers realized there was a need to design a different governance for a cultural center, taking on the mission to practice creativity and innovation collaboratively, until becoming defined as an “incubator of communities and commons”, with inclusive invitations to anyone with the knowledge, talent or enthusiasm to develop a new idea. Teams are often formed to develop projects in production workshops. At the EUt+ alliance, there is this notion that each group is an experiment in itself in team- and community-building as it blends people from different backgrounds (artistic, scientific, technical), levels of specialization (experts and beginners) and degrees of engagement. The focus of the EUt+ Citizen Lab is to produce knowledge through cooperation, collaboration, and collective cocreation. A Citizen Lab is, therefore, from the two cases described above, about creating knowledge and about creating commons, respectively “for society” and “with society.” As such, the Citizen Lab approach was described and developed within the EUt+ Horizon 2020 project “EXTRAS.” During the 3-year project, the Citizen Lab took the shape of an interdisciplinary, exploratory vehicle for eight alliance partners (France, Latvia, Germany, Bulgaria, Spain, Cyprus, Romania, and Ireland) who participated in “EXTRAS” to interact with society in a meaningful and scientific manner.

The Action Design Research (ADR) method, commonly used in the field of ICT, was chosen to serve as the central approach to be used for creating the structure and finding of artifacts (problems) within the EUt+ Citizen Lab. First introduced by Sein et al. (2011), ADR focuses on four stages: (1) problem formulation; (2) building, intervention, and evolution; (3) reflection and learning; (4) formalization of learning. In order to facilitate international engagement and establish meaningful connections across the EU, it was clear that digital elements will be paramount to bridge the communities. Therefore, the subject of digital skills was brought forward as one of the first topics to be piloted. During the summer of 2024, a pilot project connecting Latvia, France, Cyprus, Bulgaria, Germany and Spain was launched as a series of online workshops using ADR. The paper describes a brief literature review of digital skills for citizens, outlines ADR methodology, presents data and findings from the pilot workshops as well as gives conclusions on the form and outcomes of first EUt+ citizen science activities.

### 1.1 Digital skills—needs analysis

The future of Europe has a digital dimension. The European Commission has established that in Europe more than 90% of professional roles require a basic level of digital knowledge, just as they require basic literacy and numeracy skills (European Union, 2023). The use of digital tools is spreading across all sectors from business to transport and even to farming. Yet, around 42% of Europeans lack basic digital skills, including 37% of those in the workforce (European Union, 2023). The necessity and importance of digital skills has launched three programmes to help ensure the EU's digital future: (1) European Skills Agenda; (2) Digital Education Action Plan; (3) Digital Skills and Jobs Coalition. Digital skills have gained more traction since the Covid-19 pandemic in 2020. There has been an uptick in global research that connects digital skills to e-learning, the labor market and employment, study processes, and other issues (Männasoo et al., 2023; Carabregu-Vokshi et al., 2024; Arandas et al., 2024). Preliminary discussion within the working group of EUt+ Citizen Lab brought up three challenging dimensions concerning digital skills:

1. Data collection, analysis and sharing. in order to conduct citizen science across multiple EU countries, there would be a need for large amounts of data sets that would allow us to identify common problems, joint patterns, regional dynamics. in a recent organization for economic co-operation and development (OECD) report, it is outlined that there is a need for skills of advanced and data-intensive digital tools to gain insights and develop predictions (OECD, 2020).
2. Communication and interaction are essential for communities to be able to fully collaborate and engage. The same OECD report mentioned above refers to the development of digital identity and the online communication of scientific work (OECD, 2020). Therefore the need for upskilling is not only on the citizens' side, but also the scientific community's side, online communication being important aspect of digitalisation in scientific research.
3. Digital services and identity (to verify identity, preserve privacy, publish research results, submit projects, access e-services). an important point is e-governance and as discussed in the slovak republic example – the level of digital skills in country being below average, some age groups do not have sufficient digital skills to use all e-government services (Stofkova et al., 2022).

**Figure 1:**
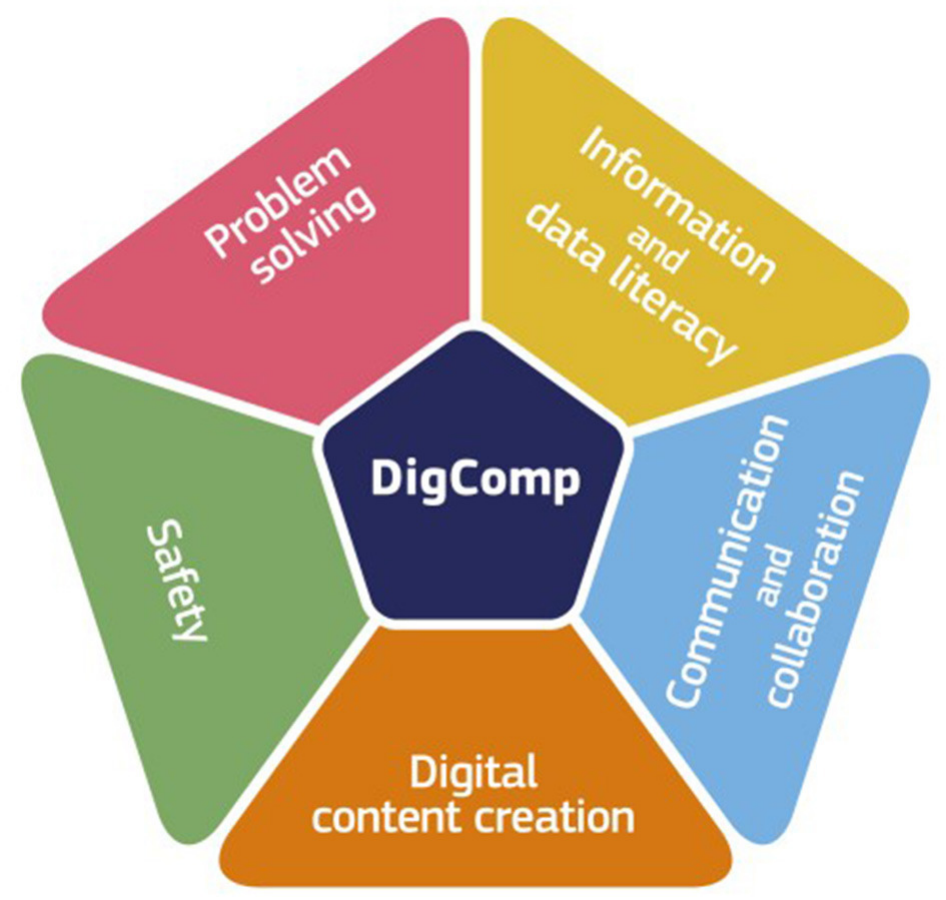
DigComp competence framework^1^.

It is often more common to refer to digital competences rather than skills where a competence is defined as “the confident, critical and responsible use of, and engagement with, digital technologies for learning, at work, and for participation in society. It is defined as a combination of knowledge, skills and attitudes,” (Council Recommendation on Key Competences for Life-long Learning, 2018). The EU has developed a comprehensive competence framework, the “Digital Competence Framework for Citizens” or DigComp, which provides a common understanding of what digital competence is (Vuorikari et al., 2022). It identifies 5 areas that citizens should possess (see Figure 1).

The factors mentioned above are just a few examples that are related to the vast landscape of digital skills. In this regard, the EUt+ Citizen Lab working group needed to address this from a broader perspective. It is also possible to assume that a fully functioning pan-European Citizen Lab would also require digital skills for experts and citizens to take part, hence the following initial research question emerged to be discussed in the beginning of the pilot workshops: “How will the growing demand for digital skills impact your community in the future?”

## 2 Methodology

Action design research (ADR) has become widely accepted as a prominent research method within information systems when managing design-oriented research projects (Cronholm and Göbel, 2022). The EUt+ (https://www.univ-tech.eu) Citizen Lab (EUt+ CL) working group has discussed a need to establish a simple, easy to replicate, easily-understandable approach that would serve as a guiding factor for citizen science. ADR provided a promising hypothesis as one such approach as it includes the formalization of learning at its final phase. This means that experts and scientists together with active citizens could convert the learning and knowledge gained during participation in EUt+ CL activities into something that can be used, exported or transferred to other contexts. Following suggestions by Petersson and Lundberg (2016) about ADR in their paper, “one possible solution optimized for the given context,” the EUt+ CL working group aligned participatory workshop design with ADR stages, while adjusting the elements of ADR with simple and easy to understand subsets of activities; for example, using phrases that emphasize challenges and opportunities instead of ‘problems.' Here is a brief outline for each of the ADR stages (as per Sein et al., 2011; Cedergren and Hassel, 2022):

1. Problem formulation - the point of departure for the ADR process is to define the problem perceived in practice. it is important to note, however, that the aim of the ADR process is not restricted to solving the particular problem *per se*, which would be the aim of a consultant, but to generate knowledge of how the class of such problems can be addressed (Sein et al., 2011). Before each workshop, EUt+ CL facilitators prepared an overview from scientific literature and global data that presented a more appropriate entry into the subject.
2. Building, intervention, and evaluation - based on the problem formulation and the theoretical underpinning laid out in the first phase, this second phase comprises an iterative process of building an artifact (valuable knowledge). in this phase, the researchers and the practitioners contribute to the development of the artifact through their knowledge and experience. Mutual evaluation runs as a parallel activity throughout this process and shapes the knowledge that emerges (Cedergren and Hassel, 2022).
3. Reflection and learning - this phase involves a continuous reflection on the design of the artifact and the need for revising the design principles. as such, the phase involves a shift from solving a specific problem to applying that learning to deal with a broader class of problems (Cedergren and Hassel, 2022).
4. Formalization of learning - the final phase involves formalization of the design principles for the class of problems that the artifact has addressed, and as such, contributes to more generalized knowledge about how similar problems may be addressed in related contexts. as highlighted by Sein et al. (2011: p. 52), the iterative process of building and evaluating the artifact in its organizational setting means that “action design researchers are well positioned to analyze the continuing adaptation of the artifact and the local practices of its use, and to make such analysis the basis for generalizing” (Cedergren and Hassel, 2022).

Taking into account available literature and context of user driven innovation needed for the Citizen Lab approach, a decision was made to adapt and implement ADR as the main methodological driver for the EUt+ Citizen Lab activities. Prior to the pilot workshops described further in this paper, small scale pilots were held with the representatives and students from EUt+ consortium members, which showed great promise for the future use of ADR.

## 3 Results

### 3.1 Pilot workshops

The pilot of the EUt+ Citizen Lab on the digital divide in professional skill development was held online on July 1, 2, and 4, 2024 via Zoom. The pilot was divided into three workshop sessions, each held on a separate day, following the ADR methodology adapted to fit the goals of the EUt+ Citizen Lab. The first three ADR phases, i.e., Problem Formulation; Building, Intervention, and Evaluation; Reflection and Learning, were addressed in separate workshops to allow for greater focus and minimizing cognitive overload for the participants. Each of the three workshops was approximately 1.5 h long. The final phase of ADR, Formalization of Learning, took place once the three online workshop sessions had concluded.

The working group selected the potential workshop participants based on their perceived interest in the topic of this Citizen Lab pilot, i.e., “Digitalization in professional development, reskilling, and upskilling.” Each working group partner invited the stakeholders they identified for whom the topic might be relevant. Using the EUt+ partnership network, twenty potential organizations and individuals interested in and working in the field of digital skills were identified and invited to participate in the pilot workshops. The potential participants identified by the working group were sent a digital informational flier that provided an overview of the Citizen Lab pilot, the potential gains from participating in the workshops, and a QR code and a link that the participants could use to sign up for the Citizen Lab. To accommodate participant schedules and ensure maximum attendance, the dates for the workshops were decided on through an event planning platform that allowed the participants to share their availability.

In the end, six participants from four countries (Latvia, France, Cyprus, and Bulgaria), representing four different organizations signed up to participate in the Citizen Lab pilot. The organizations represented were a lifelong learning center in Latvia (University of Life Sciences and Technologies Lifelong Learning Center, 2023), a third place and popular education association in France (Le Rucher Creatif, 2023), and a research and educational organization in Cyprus (Stando Ltd, 2023), and the Bulgarian Union of Standardizers in Bulgaria. Of the six participants four completed all three workshops (i.e., two participants from France, one from Cyprus, and one from Bulgaria). Original estimate for the number of participants was deemed to be from five to ten, therefore the number reached was sufficient to carry out the pilot workshops. Challenges during the recruitment of participants were related to the total amount of time required for all of the workshops, which revealed constraints among interested participants. Two participants who did not complete all of the workshops indicated that the main reason was unplanned professional commitments. This occurrence did not impact the outcomes of the study as the problem formulation phase was already concluded.

As described earlier, the pilot of this Citizen Lab focused on digital skills in professional skill development, upskilling, and reskilling. This topic was deemed to be relevant to each of the invited participating organizations in various ways: the University of Life Sciences and Technologies Lifelong Learning Center in Latvia provides educational services to adult learners to enhance employability. As the necessity for digitally skilled workers dominates the EU labor market, learning how to address the lack of digital competencies was of interest to this organization. Similarly, the Bulgarian Union of Standardizers organizes the study and supports the implementation of Bulgarian, European and global experience in the field of standardization, certification and market supervision, by conducting training paying particular attention to the use of digital skills. Among other functions, Le Rucher Creatif in France offers opportunities for digital skill enhancement for professionalization through online or in-person courses. STANDO LTD in Cyprus is focused on vocational education and training and offers courses, seminars, and consulting services.

### 3.2 Phase 1: problem formulation

The first workshop addressing the first phase of ADR, Problem Formulation, began with an introductory segment where participants expressed their reasons for attending the Citizen Lab and described their expectations for the workshops. An ice-breaking activity was used in the beginning of the session to create a fun and friendly atmosphere and encourage everyone to interact with one another. Following a brief online pre-survey, participants were introduced to key notions necessary for participation in the workshops. The notions discussed included citizen science, citizen labs, and the EUt+ Citizen Lab. Additionally, an overview of the goals and objectives for each workshop was provided to set the stage for the discussions ahead. An MS PowerPoint presentation was used to provide clarity and to lead participants through the various tasks during the workshop.

**Figure 2:**
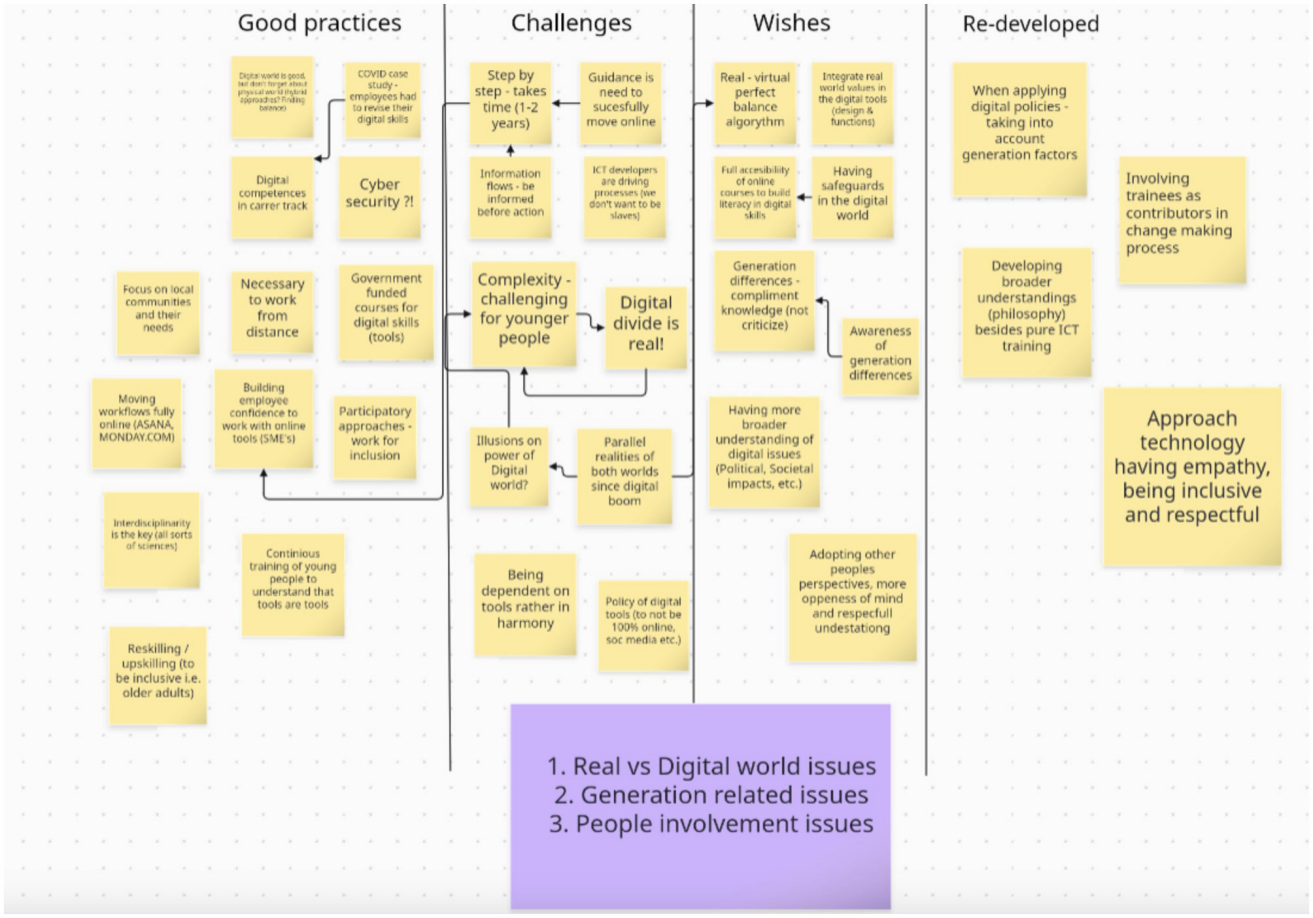
Summary of participant inputs from online whiteboard.

After the introductory portion of the workshop, the participants were divided into two breakout groups, each led by two facilitators, to explore the topic of digital reskilling and upskilling. The built-in whiteboard feature of Zoom was used to facilitate discussions in the breakout groups and take notes (see Figure 2). The participant groups were asked to begin the discussion by addressing the question “How will the growing demand for digital skills impact your community in the future?” The two groups were asked to share best practices in addressing the need for the improvement of digital skills and challenges they face with addressing this need. Additionally, the groups were asked to consider their wishes for how digital skills should be addressed more successfully within their organization. Finally, the groups were asked to consider how the best practices they shared could be re-developed to be more successful.

The groups were asked to begin with sharing best practices to set the stage for sharing positive experiences instead of immediately focusing on challenges that their organizations face. Once best practices were shared, participants were asked to consider the challenges they face. By stating the challenges, the participants identified pain points in digital skills attainment for the communities that constitute their user base (e.g., adult learners seeking to re-enter the workforce). Some of these were locally-situated issues and some were global issues faced by all participants. For example, the French and Bulgarian participants identified language barriers in teaching digital skills as an issue faced by immigrant communities. This was not an issue that was experienced by all participants, while inclusion barriers such as age, disability, and cultural barriers were acknowledged as relevant to all workshop participants.

The “wishes” portion that followed “challenges” was used to reframe the need for change in a positive way in the sense that “challenges” portion focuses on a lack while “wishes” were meant to create space for identifying opportunity in addressing the problem. The “re-developing best practices” portion of the discussion intended to facilitate reflection of the experiences shared by the participants in the group and to think through ways how best practices can be improved further to address the individual challenges identified earlier.

**Figure 3:**
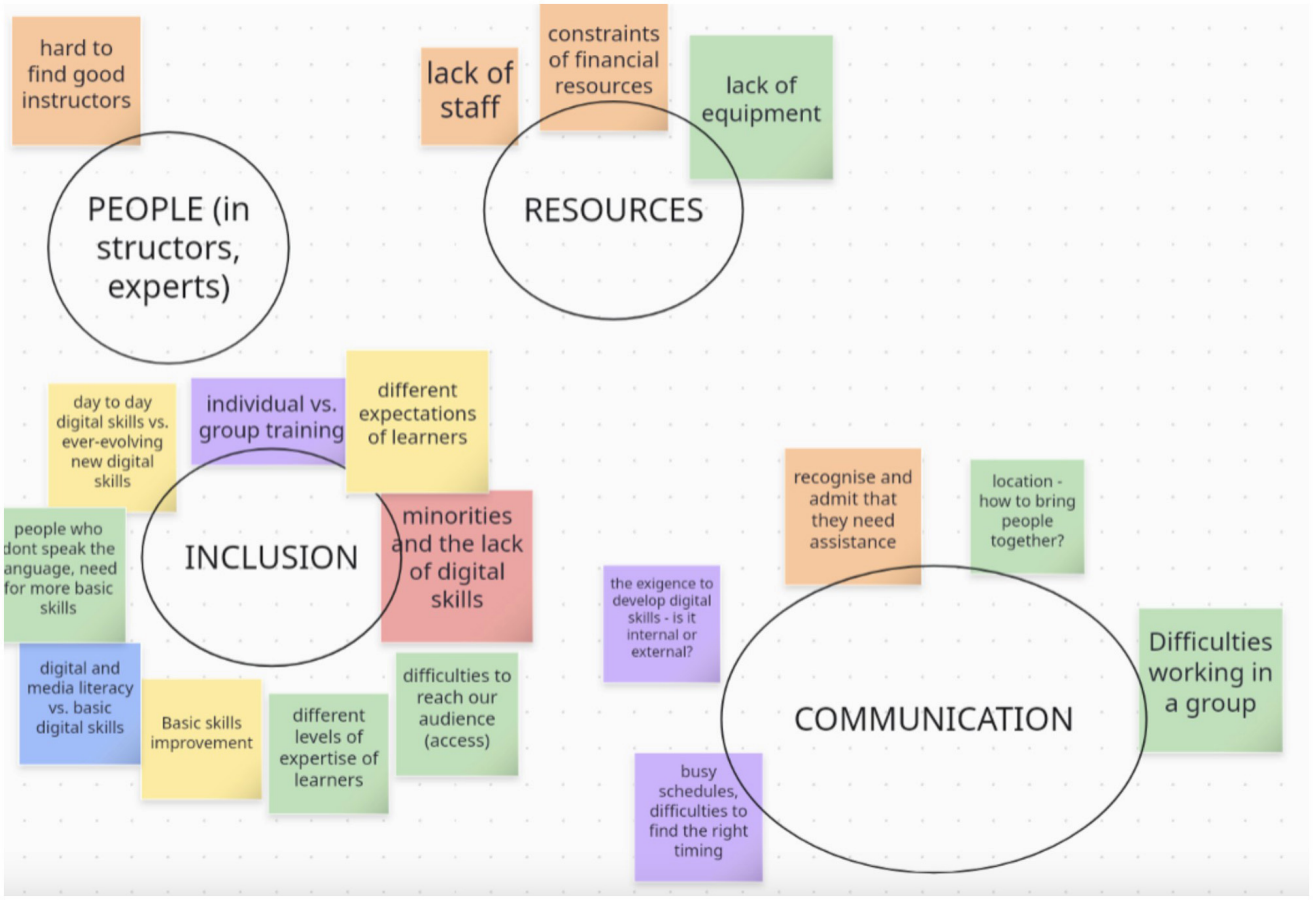
Clustered summary of participant inputs.

Finally, the groups were asked to thematically cluster the ideas shared during the discussion (see Figure 3). Clustering is an essential element of the ADR method, which enables research questions to emerge, which are then addressed in the second phase of the Citizen Lab workshops.

Once clustering of ideas was completed, both groups returned to the main room in Zoom. The session ended with a reflection, during which the groups discussed commonalities and differences in their discussions, laying the groundwork for the next workshop. The participants were also given a snapshot of what to expect in the next workshop.

The facilitators and the working group met after the workshop to share their impressions and perspectives on how the session went. Adjustments to the subsequent workshops were decided on, based on issues encountered by facilitators and the feedback of the working group members who observed the workshop. Additional help with the Zoom whiteboard from the secondary facilitator, the topic clustering procedure, and timekeeping were among the most commonly shared issues that participants felt needed to be addressed.

### 3.3 Phase 2: building interventions

The second stage of ADR was addressed the following day at the second Citizen Lab workshop. After another icebreaking activity, a recap of the previous workshop and its outcomes was presented to everyone, along with an overview of the plan for the second workshop and its objectives. Due to the small number of attendees (four returning participants), it was agreed upon ahead of time that during this second workshop, all participants would stay in the main room and participate in the activity as one group rather than be split into two breakout groups.

Six research questions emerged from the discussions and ideas shared during the first workshop. The questions were formulated by the Citizen Lab working group and presented to the participants for review and discussion. These questions were proposed as a starting point for the discussion to cover a wide range of subjects and explore various theme combinations. The questions were formulated based on the thematic clustering activity that concluded the breakout group activities during the first workshop. Participants were invited to reflect on these questions and to offer comments and suggestions. They reformulated the questions together until they satisfactorily reflected their shared concerns and experiences. Initially offering the participants “draft” research questions to work with meant to serve as a catalyst for discussion. Co-creating and re-shaping the research questions ensured that the participants had not been misunderstood or their viewpoints misrepresented by the facilitators and the working group.

Next, three out of the six questions were selected as the most relevant to all participants. Then, the scope was reduced even further as the participants were asked to negotiate and choose one of the three research questions to focus on in-depth. The question the group ultimately chose to address was “How do we welcome everybody as they are, while developing digital skills?” This question was related to inclusion barriers that participants identified through their experiences with facilitating digital skills development. The participants expressed that while helping individuals develop digital skills, there is a need to create more acceptance, respect, and space for people's identities and individual challenges.

**Figure 4:**
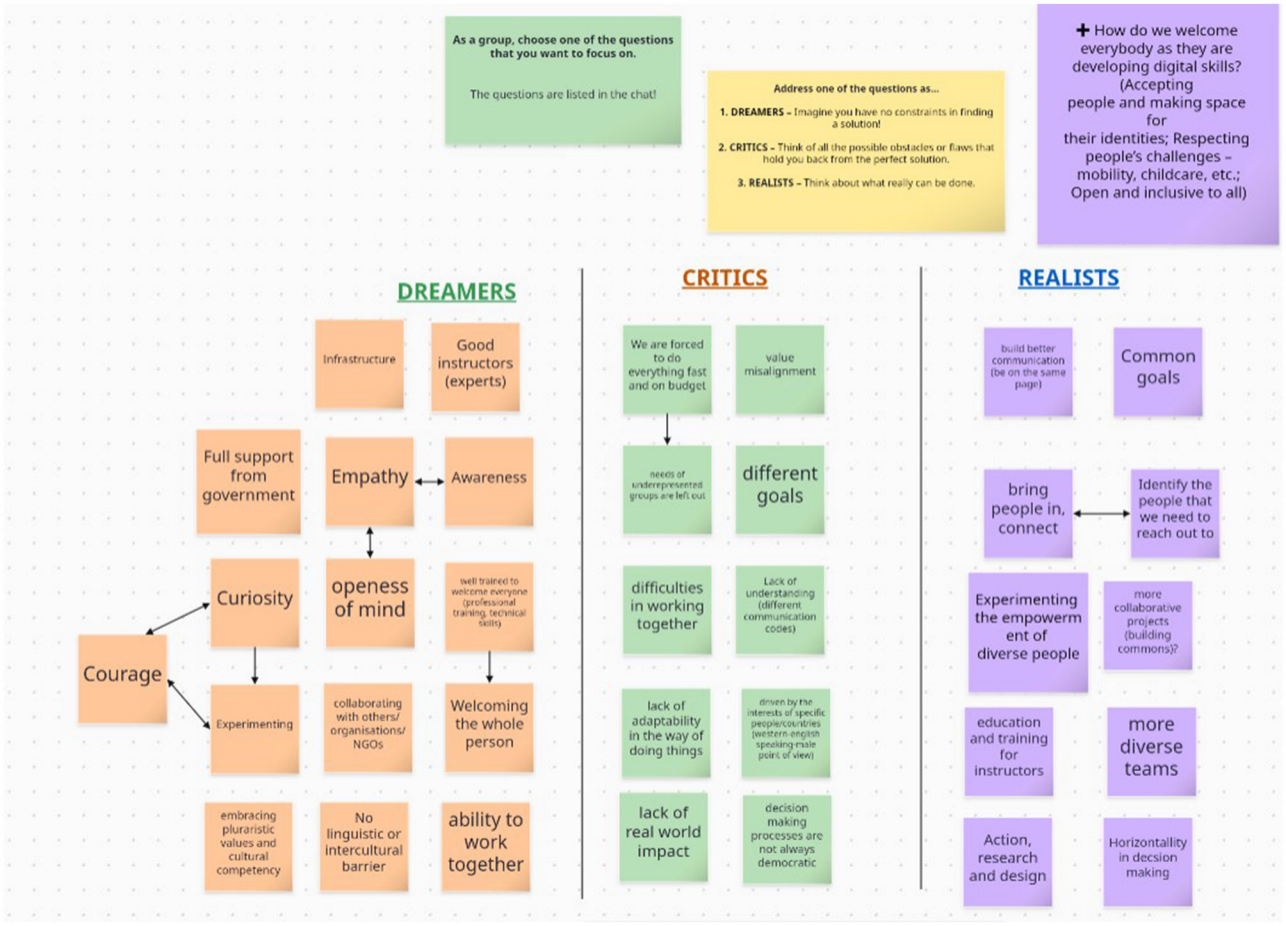
Summary of participant inputs for phase 2.

The participants were asked to consider the research question from three distinct perspectives, adapted from the Design thinking methods catalogue (2023) as dreamers, realists, and critics. This method encouraged a thorough examination of the question at hand, allowing participants to envision ideal scenarios, evaluate practical realities, and identify potential difficulties in addressing the issue (see Figure 4).

The session concluded with a recap of the key points discussed in the session and a preview of what the participants could expect from the final workshop to come.

### 3.4 Phase 3: reflection and learning

At the beginning of the third and final day of workshops, participants were given a summary of the work accomplished during the previous two workshops. This overview helped refresh the participants' memory and set the stage for the upcoming activities during the final workshop.

**Figure 5:**
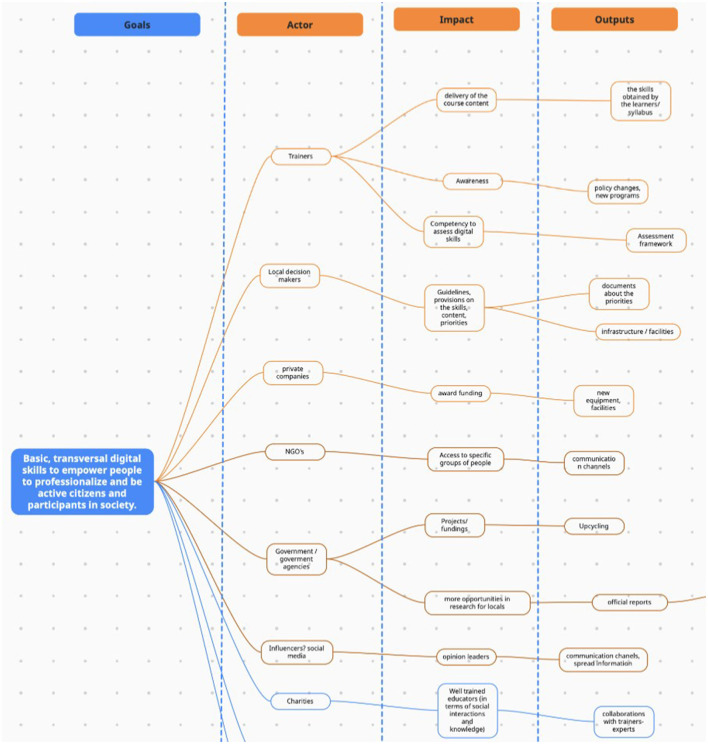
Summary of participant inputs for phase 3.

Following the summary, participants engaged in a group activity focused on the research question selected during the second workshop, “How do we welcome everybody as they are, while developing digital skills?” The task was to collaboratively transform this research question into a goal. It was necessary to reframe the research question into a goal to move onto identifying the primary actors involved in the processes that surround the issue identified through the research question. To contemplate what it would take to achieve the goal, “Basic, transversal digital skills to empower people to professionalize and be active citizens and participants in society,” the participants had to assess the potential impact of the actors, outline the actual steps required, and outputs produced by the actors (see Figure 5). Building on this foundation, participants were invited to collaborate on creating a plan for achieving the goal. By identifying the key players involved in processes connected to the goal and their influence, the group could explore intersections of activities or impacts that might provide valuable insights into what actions are needed to move closer to the goal attainment.

The session concluded with discussions on what steps can be taken to implement the action plan. The participants were asked to give their feedback on their experience during the Citizen Lab through an anonymous post-workshop survey. The post-workshop survey provided valuable feedback on the process, the workshops, and their content. Each participant was informed that they would receive a certificate of participation as well as an acknowledgment in a publication.

## 4 Discussion

### 4.1 Discussion of the results of the workshops

The discussions that took place during the Citizen Lab workshop sessions proved to be fruitful, with participants delving deeply into the topic of digitalization in professional development, digital upskilling and reskilling. The discussions during the three workshops gave useful insights to both the participants and the researchers into the multifaceted nature of the issue of digital skills in the landscape of Europe's labor market. While it was evident that the experiences the participants shared were bound to local contexts and issues pertaining to the specific countries they represented, the participants were able to relate to each other with ease. The issues pertaining to inclusion when teaching and learning digital skills were particularly relevant to all participants. Some participants expressed that, in their experience, adult learners face difficulties with developing their digital skills due to age while others emphasized the learners' caregiving responsibilities that limited their time to devote to digital upskilling or reskilling. For others, cultural and language barriers, especially in the case of immigrant learners or minorities, were of great importance when considering digital skill attainment. Sensitivity to people with disabilities was also seen as lacking by many in local programs targeting digital skill improvement.

The overall takeaway from the three sessions was that it is necessary to develop programs targeting digital skill attainment with inclusion and the intersectionality of identities in mind. Instructors are often ill prepared to work with adult learners who face a variety of obstacles related to inclusion. However, the conversations that emerged during the Citizen Lab also highlighted a focus on the challenges and doubts around the real-world implementation of the theoretical concepts addressed and knowledge gained. The participants had concerns that the Citizen Lab workshops might not lead to tangible outcomes that would address the problems discussed, which is a common issue with similar events that often remain fixed at a theoretical level. This concern was also reflected in the post-survey with some participants noting that the third Citizen Lab workshop, which focused on mapping the action plan lacked more concrete steps toward implementation. That being said, the researchers and participants agreed to collaborate on relevant Erasmus+ project proposals that would address the issues identified during the Citizen Lab workshops in the future as one path toward implementing the action plan.

Despite the valuable insights provided by participants, the data and feedback were somewhat limited due to the diverse levels of experience and expertise among the attendees. Additionally, some participants did not align well with the Citizen Lab's focus, which limited the depth and quality of the discussions at times. While the participants who were invested in the topic of digital skills were actively engaged, those participants who could not find clear links between the topic and their daily work could not contribute as readily.

The results of the post-survey indicate that participants improved their understanding of the current landscape in digital skills in Europe and on citizen science compared to their initial knowledge of the subjects. This improved comprehension suggests that the EUt+ Citizen Lab workshops were successful in explaining key concepts and principles of citizen science. However, despite this increased understanding and awareness, the participants remain unsure about the impact of citizen science on real-world situations. This shows that while the workshops succeeded in establishing a core knowledge base, more concrete examples are required to demonstrate how citizen science can lead to meaningful change in practice.

Participants expressed a range of preferences regarding which workshop they found most enjoyable, with particular emphasis on the first and third session. Workshop 1 was commended for its engaging exchange of experiences, in which the participants had the chance to share views and learn about the reality of digital inclusion in other EU countries. Workshop 3 was lauded for its practical focus. Interestingly, these two workshops were also mentioned as the ones that need improvement, indicating that while they were valuable, there is still room to enhance the workshop structure and content. For example, for Workshop 3, the participants underlined the need for clearer questions that would result in more tangible outcomes, stating that while the ideas presented were useful, the session did not feel sufficiently precise to ensure effective implementation of any actionable steps.

### 4.2 Reflection and recommendations for future implementation

The completion of the EUt+ Citizen Lab workshops on digitalization in professional development, digital upskilling and reskilling have provided valuable insights on both the topic and on using the Action Design Research methodology for the purposes of citizen science activities. The Citizen Lab revealed both successful strategies and areas for improvement in engaging individuals in productive experience sharing and knowledge co-creation activities to unearth artifacts that can lead to solution-seeking activities. Analyzing the outcomes and feedback from these meetings led to several important implications for future Citizen Lab design and execution in terms of effectiveness, engagement, and impact.

#### 4.2.1 Enhanced participant selection and suitability

Implementing a more specific Citizen Lab participant selection procedure would guarantee that participants have sufficient expertise, experience as well as an interest in the subject. This strategy will help ensure that participants are not just aware, but also actively involved and invested in the discussions surrounding the topic at hand. By connecting participants' backgrounds with the workshop's aims, the quality of discussions improves, making contributions more relevant and leading to more significant outcomes overall. It is necessary to ensure that participants have a clear understanding of the goals and objectives of the Citizen Lab as well as the expectations of their contributions during the workshops, so that they can make a well-informed decision of whether this type of event is suitable and relevant to them.

#### 4.2.2 Time management, facilitation, and group dynamics

The Citizen Lab working group anticipated that 1.5 h would be sufficient for each of the three workshops that constituted the Citizen Lab. Though the participants managed all of the tasks that were planned in each workshop, it is evident that more time per workshop – 2 h, is optimal. This is particularly the case with Workshop 1 where the introductions and theoretical discussion takes up a significant amount of time. Notably, this Citizen Lab began with six participants present in the first workshop and the time needed for each participant to introduce themselves, their organization and their connection to the topic of the Citizen Lab, however brief, was significant (approximately 15 min for this segment alone). The theoretical background that provided context to the participants on citizen science, citizen labs, and digital skills in the EU labor market took up more time than originally anticipated. If more participants are included in future Citizen Lab workshops, the organizers must account for a longer introductory segment.

Besides this, there were several workshop segments that took longer than expected. During Workshop 2, the participants took much longer to reformulate and decide on the research question they would like to focus on in-depth, which cut some of the time originally planned for the next activity. That said, the discussions surrounding the research question proved to be immensely interesting and gave the researchers a better understanding of the main areas of concern for the participants, which underlined the importance of this activity.

This discussion surrounding the proposed research question also illuminated the stark difference in approaches between practitioners (the participants) and academics (the Citizen Lab working group researchers who actively participated in the sessions to facilitate discussion). While the Citizen Lab working group engaged in a lengthy, in-depth discussion of the use of precise language in the formulation of the research question (e.g., “What do we mean when we say ‘citizens'?”), the participants were less preoccupied with the minute details of wording and tried to ground the conversation in practical application of the research question. This signaled to the researchers that while their involvement in the discussions is necessary as the ADR method supports guided emergence of artifacts, academics should take care not to overwhelm the participants with in-depth theoretical considerations. The facilitators must ensure that all of the parties involved in the discussion stay on topic and move toward the goal of the activity.

In an online setting, group dynamics can be challenging to manage, especially when the participants are unknown to the organizers. Facilitators must ensure that each participant feels that their voice matters and that their experiences are valuable for the workshop. As mentioned earlier, due to misalignment of some of the participants and the topic of the Citizen Lab, some were more reluctant to speak up during the discussions. Even when invited to contribute, some participants declined to add to the discussion at times. In the future, the organizers of such Citizen Labs must consider not only how to find the participants best suited for the topic, but also how to best encourage active participation in the case where participants feel reluctant to share their thoughts. The aspect of language barriers should be considered here. None of the participants were native English speakers. The facilitators felt that this also played a role in the willingness to contribute for some participants. Moreover, as some participants were very active and engaged, others may have felt somewhat overwhelmed. It is necessary to consider mitigation strategies to alleviate participant anxiety and consider what may hinder their active participation.

#### 4.2.3 Connecting theory to practical implementation

Addressing the disparity between academic and theoretical discussions and practical applications is critical for future Citizen Lab workshops. Participant feedback showed that, while the workshops provided useful theoretical insights, there was a lack of emphasis on how these principles could be practically applied. In order to bridge this gap, subsequent sessions ought to include real-life examples, case studies, and tangible initiatives.

It is clear that the ADR method emphasizes the emergence of artifacts and the formalization of learning with the added importance of knowledge co-creation in its application in a Citizen Lab, which is to say that a practical solution to a given issue is not the goal of the Citizen Lab in this format of three workshops. Certainly, a practical implementation plan for a concrete solution is difficult to achieve under the time constraints of this type of an event. This points to the need to manage participant expectations and to ensure that all parties involved understand what can be achieved and what is beyond the scope of the Citizen Lab from the get-go. Otherwise, participants may end up feeling disappointed and discouraged from participation in similar initiatives in the future.

Still, it is necessary to consider how to move from the theoretical and exploratory nature of the Citizen Lab to a plan with actionable steps that follow the knowledge co-creation for future iterations of the Lab. It is important for organizers to consider the value proposition to the participants invited to contribute to the Citizen Lab – dedicating 1.5–2 h of time and energy is no small request. Moreover, while the researchers gain invaluable theoretical insights and data, the participants may not feel that they receive equal benefits for their involvement. Experience and knowledge exchange is no doubt valuable, but as discussed previously, participants desire concrete, real-life applications and impact of the work they do during the workshops. This point must be considered carefully and possibilities for actionable steps to follow the Citizen Lab should be explored by the organizers as part of the workshops. For this, there may be a need for a fourth Citizen Lab workshop with a distinct practical implementation focus.

## 5 Conclusions

The concept of an internationally functioning Citizen Lab still holds value even after 3 years after the launching of the EUt+ alliance Horizon 2020 project “EXTRAS”. Advancement in direction of Citizen Science has been made and with the concept of ADR and participatory workshops, it seems as a feasible solution to the needs that were brought forward. Digital skills being one of example subjects, it was possible to approach subjects with a common interest and align discussions using ADR framework. Building on previous experience of the Citizen Lab working group, it was possible to prepare and facilitate workshops in an efficient manner.

The three Citizen Lab pilot workshops fulfilled their goal of testing the applicability of the Action Design Research methodology for the purposes of citizen science activities. The ADR method provided a clear structure for the Citizen Lab, which helped participants engage in meaningful discussions surrounding the topic of digitalization in professional development, digital upskilling and reskilling in the context of the EU.

The activities held during the Citizen Lab workshops facilitated an international experience exchange between four practitioners who completed all three workshops from three different countries (France, Cyprus, and Bulgaria) and the researchers in the Citizen Lab working group of the European University of Technology (EUt+). The three Citizen Lab workshops lead to an emergence of ideas of what gaps must be addressed in fostering digital skills development for individuals in various adult education contexts. The main finding shared by all participants was the need for more inclusive approaches to digital skill development, making space for various identities and centering intersectionality.

Using international collaboration, the ADR framework and a vast digital skill subject area, it has been possible to perform exploratory discussions, cluster ideas, and generate design principles. The authors of this paper argue that this serves as a proof of concept for internationally situated Citizen Labs performing meaningful and practical citizen science. It is possible to apply the same methodology in different contexts (in terms of subject area) and different formats (in terms of onsite, virtual or hybrid), with the methodological caution that outcomes and artifacts cannot be predicted, since they are anchored in the participant expertise and interest pool. The overarching aim of the concept and pilot was to test and observe an approach elaborated by the Citizen Lab working group and with that it is deemed successful.

## Data Availability

The raw data supporting the conclusions of this article will be made available by the authors, without undue reservation.

## Footnotes

^1^ https://joint-research-centre.ec.europa.eu/scientific-activities-z/education-and-training/digital-transformation-education/digital-competenceframework-citizens-digcomp/digcomp-framework_en
